# Metabolomic Profiling and Antioxidant Activity of Fruits Representing Diverse Apple and Pear Cultivars

**DOI:** 10.3390/biology10050380

**Published:** 2021-04-28

**Authors:** Mauro Commisso, Martino Bianconi, Stefania Poletti, Stefano Negri, Francesca Munari, Stefania Ceoldo, Flavia Guzzo

**Affiliations:** 1 Biotechnology Department, University of Verona, Strada Le Grazie, 15, 37134 Verona, Italy; mauro.commisso@univr.it (M.C.); stefania.poletti@univr.it (S.P.); stefano.negri@univr.it (S.N.); francesca.munari@univr.it (F.M.); stefania.ceoldo@univr.it (S.C.); 2 Demethra Biotech Srl, Via dell’Innovazione, 1, Camisano Vicentino, 36043 Vicenza, Italy; martinobianconi@dembiotech.it

**Keywords:** apple, pear, fruit polyphenols, antioxidants, untargeted metabolomics

## Abstract

**Simple Summary:**

Apples and pears are popular with consumers because of their pleasant taste and high content of bioactive, health-promoting phytochemicals. However, the metabolic profiles of apples and pears are affected by genetic and environmental factors and, therefore, differ even between cultivars of the same species. Biochemical characterization and testing of antioxidant activity are useful tools to identify cultivars with superior organoleptic properties and potential health benefits. We, therefore, used these complementary approaches to analyze the metabolic profiles of six apple and five pear cultivars during two growing seasons, revealing variations in the content of primary and specialized metabolites. We found one ancient Italian apple cultivar rich in polyphenols and one pear cultivar low in sucrose that may be particularly attractive to consumers.

**Abstract:**

The false fruits of apple (*Malus domestica*) and pear (*Pyrus communis*) are consumed all over the world, contributing to the dietary intake of health-promoting antioxidant phytochemicals. For example, polyphenols confer many beneficial effects (according to their chemical structure, bioavailability, and absorption efficiency in the gut) and the consumption of polyphenol-rich apple and pear fruits may therefore reduce the risk of some diseases. However, the content of such molecules is highly dependent on the specific fruit cultivar. To examine this metabolic diversity in detail, we used metabolomic analysis (NMR and HPLC-DAD/MS) to profile the metabolome of six apple and five pear cultivars. We also determined the antioxidant capacity of the extracts (FRAP assay) and correlated this with the metabolomic composition and abundance of specific metabolites. We observed the cultivar-specific accumulation of sugars, amino acids, malic acid, and various polyphenols, which was also related to the growing season for some cultivars. We found that the ancient Italian apple Pom Prussian was enriched for chlorogenic acid as well as more characteristic polyphenols (phloretin derivatives), the pear cultivar Abate Fetel was low in sucrose, and both cultivars displayed high in vitro antioxidant activity. These cultivars may, therefore, be particularly attractive to health-conscious consumers.

## 1. Introduction

The pomaceous false fruits of *Malus domestica* (apple) and *Pyrus communis* (pear), both representing the Pomoideae subfamily of the Rosaceae, are widely consumed and appreciated worldwide, with production expected to reach 76.1 million tonnes of apples and 22.2 million tonnes of pears in 2020/2021 [1]. Both fruits provide a good source of dietary fiber and potassium [2]. They are also rich in polyphenols: apples accumulate hydroxycinnamic acids (chlorogenic acid), flavan-3-ols, procyanidins, flavonols (quercetin), dihydrochalcones (phloretin) and (in red-peel cultivars) anthocyanins [3]; pears mainly accumulate hydroxycinnamic acids, flavan-3-ols, procyanidins, flavonols (quercetin and isorhamnetin) and (in red-peel cultivars) anthocyanins [4,5]. As recently reviewed [6], these metabolites exert antioxidant activity both on human after fruit consumption and on fruits, that accumulate them in order to keep their REDOX homeostasis.

Although other fruits accumulate more secondary metabolites than apples or pears, and display higher antioxidant activity, the widespread consumption of apples and pears makes them an important source of antioxidant polyphenols. This is particularly true for apples, which are the most common dietary source of phenolic compounds and antioxidants in northern Europe and the USA. Various health-promoting activities have been attributed to apple and (to a lesser extent) pear polyphenols. In addition to their antioxidant activity, apple polyphenols have been shown to alleviate the symptoms of chronic non-communicable diseases, lower the risk of cardiovascular disease and cancer and promote a healthy gut microbiome [7,8,9,10,11]. Pear polyphenols have not been studied in so much detail, but antioxidant, anti-inflammatory and anti-proliferative activities have been demonstrated in vitro [2], and they may improve the regulation of blood pressure and vascular function in middle-aged and older men, and in women with metabolic syndrome [12,13].

The metabolic profiles of apples and pears depend on genetic and environmental factors, and therefore differ between cultivars of the same species and even between different orchard environments. Metabolomic characterization could help to map this metabolic diversity and facilitate the selection of healthy cultivars by breeders and consumers. However, as previously shown for the sweet cherry metabolome, a robust sampling plan is required to ensure the material selected for metabolomic analysis is representative [14]. Moreover, sample preparation, extraction, and analysis rely on different procedures and techniques, thus making it difficult to compare metabolomic data across different studies.

Here we established a robust sampling plan to investigate the metabolome of six apple and five pear cultivars in a representative manner. This involved the sampling of 50 independent fruits for each pool of trees (biological replicates), assessing the ripening stage of the fruits by measuring the pH and sugar content (°Brix), sampling in two independent growing seasons, and sampling in various orchards. Moreover, we used three complementary approaches to carefully profile the metabolome: nuclear magnetic resonance (NMR) spectroscopy for primary metabolites, high-performance liquid chromatography with diode array detection (HPLC-DAD) for quantitative targeted analysis of the major secondary metabolites, and HPLC with electrospray ionization mass spectrometry (HPLC-ESI-MS) for untargeted metabolomics. We also measured the antioxidant activity of all the samples using an in vitro ferric ion reducing antioxidant power (FRAP) assay and determined the relationship between the antioxidant activity and metabolic profiles of each cultivar.

## 2. Materials and Methods

### 2.1. Plant Material

Apple fruits from six *Malus domestica* cultivars (Fuji, Golden Delicious, Royal Gala, and Granny Smith as global varieties, Imperatore as an Italian cultivar, and Pom Prussian as a local variety) were collected from 12 orchards for commercial production located in the Veneto Region of Italy. The fruits were collected by the producers according to their readiness for marketing. Pear fruits from five *Pyrus communis* cultivars (Abate Fetel, Kaiser, Conference and Williams as global varieties, and Decana del Comizio as an Italian cultivar) were collected from 11 orchards also in the Veneto Region. Fruits were collected during the 2014 and 2015 growing seasons.

### 2.2. Sampling Plan

At least 200 fruits were collected from up to 20 different trees growing in different rows and at different positions in the orchard. The fruit selection was based on the visible maturation degree (color) and shape. Fruits were placed in four labeled boxes and, following transfer to the laboratory, those cultivated in different orchards but belonging to the same cultivar were mixed in equal proportions to create three pools (corresponding to three biological replicates) of 50 fruits (apples or pears) for metabolomic analysis. Another 30 fruits were selected for quality analysis (°Brix, pH and hardness).

### 2.3. Sample Preparation

For metabolomic analysis, apple and pear fruits were cut into halves and the true fruit parts (core and seeds) were removed using a small spoon (Schnitzmesser-set Professional; Triangle, Solingen, Germany). From each half, we prepared four equal pieces including the exocarp (skin) and mesocarp (pulp) using a professional tool for fruit sculpture (Schnitzmesser-set Professional). Two pieces were frozen in liquid nitrogen and stored at −80 °C as technical replicates. The other two pieces were frozen in liquid nitrogen and powdered using an A11 basic analytical mill (IKA-Werke, Staufen, Germany) and stored at −80 °C. For quality analysis, the °Brix, pH and hardness were determined for 30 fruits as previously described [14]. Briefly, the hardness was measured with a PCE-PTR 200N sclerometer (PCE Instruments, Lucca, Italy) at four fruit positions, then six pools of five fruits were created and the juices were extracted with a mixer. A micropH 2001pH meter (Crison, Carpi, Italy) and a DBR 35 digital refractometer (Kingstic, Ningbo, China) were used to assess the pH and °Brix, respectively.

### 2.4. NMR Spectroscopy

Sample preparation and NMR spectroscopy were carried out as previously described [14]. Briefly, we thawed 2–3 g of the fruit frozen powder and then vortexed and sonicated the resulting juice for 15 min in a cold-water bath. The soluble aqueous extracts were obtained by two steps of centrifugation at 4 °C, the first for 10 min at 15,000× *g*, and the second for 20 min at 18,000× *g*. Then, 0.56 mL of the obtained solution were diluted to a final volume of 0.7 mL containing 5% D_2_O (Cambridge Isotope Laboratories, Cambridge, UK), potassium phosphate buffer 0.15 M, 4,4-dimethyl-4-silapentane-1-sulfonic acid-d6 (DSS-d6 from Sigma Aldrich, St. Louis, MO, USA) 1 mM, and 0.02% sodium azide, and the pH corrected to pH 6. The ^1^H-NOESY spectra of the fruits soluble extracts were acquired using 64,000 data points, 10 s of recycle delay, 20 ppm of spectral width, 64 scans and a mixing time of 100 ms. Measurements were done at 25 °C with a Bruker Avance III spectrometer equipped with a triple resonance TCI cryogenic probe and operating at a ^1^H Larmor frequency of 600.13 MHz. Spectra were processed with Topspin v3.2 (Bruker). Metabolites were identified using the Profiler module of Chenomx NMR Suite v8.0 (Chenomx, Edmonton, AB, Canada) and spectra of standard compounds found in the Biological Magnetic Resonance Bank (http://www.bmrb.wisc.edu/ (accessed on 1 April 2020)). Metabolites were quantified by signals integration using the DSS-d6 as the internal reference. Concentration values were then converted to milligrams per 100 g of fresh fruit based on the starting weights and volumes.

### 2.5. Metabolomic Analysis

Targeted (HPLC-DAD) and untargeted (HPLC-ESI-MS) metabolomic analysis was carried out as previously described [14] with modifications. In the extraction protocol, three volumes of cold LC-MS-grade methanol (Honeywell, Charlotte, NC, USA) was used to extract metabolites from ~300 mg of frozen powder. Samples were vortexed for 30 s, sonicated at 40 kHz for 15 min in an ultrasonic bath (Falc Instrument, Bergamo, Italy) and centrifuged at 18,000× *g* for 10 min at 4 °C. Supernatants were diluted 1:3 (*v*/*v*) with LC-MS waters (Honeywell), filtered with Minisart RC4 filters (pores of 0.2 μm; Sartorius, Göttingen, Germany) and 30 μL were injected for the HPLC-DAD analysis, whereas 20 μL were injected for the HPLC-ESI-MS analysis. The HPLC-DAD instrumentation consisted of an autosampler System Gold 508, an HPLC Gold 126 Solvent Module and a detector Gold 168 Diode Array Detector (all provided by Beckman Coulter, Fullerton, CA, USA). The chromatographic solvents were A: water acidified with formic acid (0.5%, *v*/*v*) plus acetonitrile (5%, *v*/*v*); B: acetonitrile (100%, *v*/*v*). The HPLC method started from 0 to 10% B in 2 min, from 10 to 20% B in 10 min, from 20 to 25% B in 2 min, from 25 to 70% B in 7 min, isocratic for 5 min at 70% B, from 70 to 90% B in 1 min, isocratic for 4 min at 90% B and from 90% to 0% in 1 min and an isocratic step of 18 min. The column was an Alltima HP C18 column (150 mm × 2.1 mm, particle size 3 μm) provided of a C18 guard column (7 mm × 2.1 mm) both of Alltech Associates (Derfield, IL, USA). The flow rate was set at 0.2 mL/min. HPLC-DAD data were recorded and processed by using 32-Karat Software. Metabolite were quantified by performing calibration curves with the following authentic commercial standards: chlorogenic acid, *p*-coumaric acid, kaempferol, phloretin, phloridzin, cyanidin 3-O-glucoside (all from Extrasynthese, Genay, France), and quercetin (Sigma-Aldrich, St Louis, MO, USA). Having ensured the accuracy of the calibration curves (R^2^ > 0.997), metabolites were quantified by measuring absorption at 320 nm for chlorogenic acid and *p*-coumaric acid, 350 nm for quercetin, isorhamnetin, and kaempferol, 520 nm for cyanidin 3-O-glucoside, and 280 nm for phloretin and phloridzin. Chlorogenic and neochlorogenic acids were quantified as chlorogenic acid equivalents, *p*-coumaroyl quinic acid as *p*-courmaric acid equivalents, quercetin and isorhamnetin derivatives as quercetin equivalents, anthocyanins as cyanidin-3-O-glucoside equivalents, and phloretin 2′-O-xyloglucose as phloretin equivalents.

The HPLC-ESI-MS combined an autosampler System Gold 508 and a HPLC Gold 126 Solvent Module (both of Beckman Coulter) that were on-line with an ion trap Esquire 6000 mass spectrometer (Bruker Daltonics) equipped with an ESI ion source. The chromatographic solvents, methods and column were the same described for HPLC-DAD. ESI parameters were the following: 50 psi at 350 °C for the nebulizing gas (nitrogen) and 10 l/min for the drying gas (nitrogen). Samples were ionized in alternate mode (positive and negative), with a scan range of 50–1500 Da and a target mass of 400 *m*/*z*. Ions were fragmented by using 1 V and helium was used to perform Collision Induced Dissociation (CID).

### 2.6. LC-MS Data Processing and Metabolite Annotation

LC-MS data processing, including peak extraction, alignment and creation of the data matrix using MZmine (http://mzmine.sourceforge.net (accessed on 5 June 2020)), and metabolite identification were carried out as previously described [15]. Metabolite identification was confirmed by analyzing some samples by ultrahigh-performance liquid chromatography with high-resolution mass spectrometry (UPLC-HRMS) [16], which consisted of an Acquity I class UPLC (Waters), equipped with a BEH C18 column (100 mm × 2.1 mm, particle size 1.7 μm, a PDA detector (Waters) and a Xevo G2-XS qTOF (Waters), thus distinguishing quercetin 3-O-glucoside from quercetin 3-O-galactoside, for example.

### 2.7. Antioxidant Assays

Antioxidant activity was assessed in vitro using a FRAP assay as previously described [14].

### 2.8. Statistical Analysis

The apple and pear agronomic data were validated by performing a T-test (*p* < 0.01). LC-MS data matrices, including the relative abundance of each signal (metabolite) in each sample, were submitted for unsupervised (principal component analysis, PCA) and supervised multivariate statistical analyses (orthogonal partial least squares discriminant analysis, OPLS-DA) by using SIMCA 13.0 (Umetrics) as previously described [17]. The metabolite abundance values reported in the datamatrices were mean centered and Pareto converted before the analysis. The OPLS-DA statistical models were validated by performing 200 permutations on a PLS-DA model having the same components and a CV-ANOVA test (*p* < 0.01). Moreover, the antioxidant activity of each sample was used to create a Y matrix and its correlation with the metabolites (X matrix) was investigated by OPLS-DA and/or orthogonal (bidirectional) partial least squares discriminant analysis (O2PLS-DA). For the metabolites quantified by HPLC-DAD and the FRAP assay, each value represents the mean of three biological replicates and the error bars indicate standard deviations. To validate the HPLC-DAD and FRAP results, ANOVA followed by a post-hoc Student Newman-Keuls (SNK) multiple comparison test was performed with a level of significance of 0.05.

## 3. Results

### 3.1. Soluble Sugars and Acidity Are Mainly Cultivar-Dependent in Apple but Season-Dependent in Pear

Mature apple and pear fruits from 1–7 orchards (depending on the cultivar) were mixed in equal proportions to obtain pools of 50 individual fruits per cultivar, each one equally representative of the various orchards. This number was selected on the basis of preliminary experiments in other fruits, through HPLC-DAD analysis of extracts from pools of fruits of different size, showing that 50 fruits were as representative as 100 fruits, i.e., there were not statistically significant differences in composition between individual pools, while 25 fruits were not representative. For each cultivar, we prepared three independent pools corresponding to biological replicates. The procedure was repeated for two growing seasons (2014 and 2015).

Each pool was processed by removing the true fruit and cutting the remaining tissue (peel and pulp) into four pieces, which were frozen in liquid nitrogen, powdered, and carefully mixed. This eliminated the variability of individual fruits, trees, and orchards, but retained the common representative characteristics of each cultivar in the specific geographic zone and in the specific growing season.

**Figure 1:**
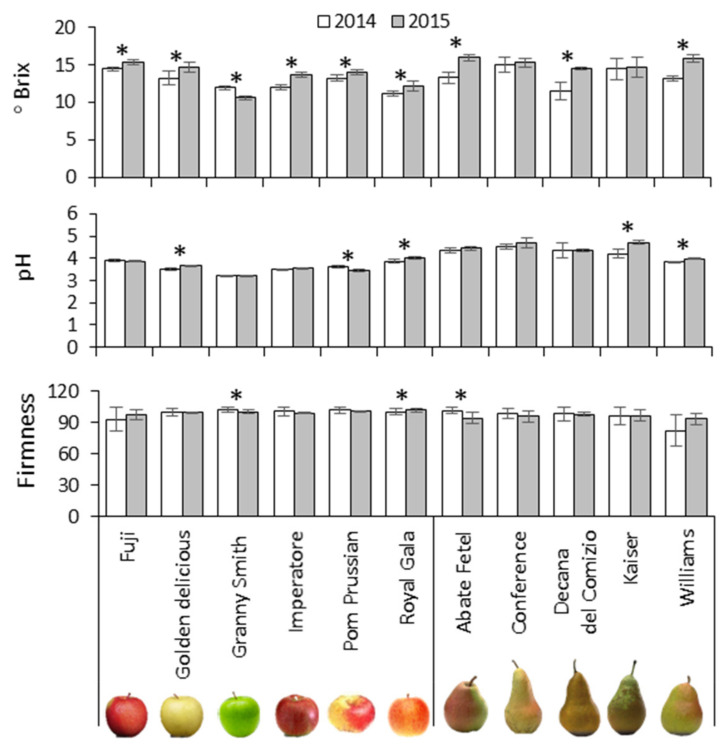
Agronomic features of the fruits from different apple and pear cultivars. Data are means of n = 30 individual fruits ± standard deviations. Significant differences between means of the two growing seasons for each variety are marked (*) for *p* < 0.01 according to Student’s *t*-test.

The fruits were collected at the stage of commercial maturity based on the phenological stage and information from the producers. To confirm the correctness and homogeneity of the maturity stage in the two vintages, we measured the pH, °Brix, and hardness of the fruits in independent batches of 30 specimens (Figure 1).

The pH of the fruit flesh was strongly cultivar-dependent both in apples and pears, with the growing season showing a negligible influence. The soluble sugar content (°Brix) was cultivar-dependent in apples but season-dependent in three of the five pear cultivars. When flesh pH, °Brix and hardness were combined (500 apples and 545 pears) for principal component analysis (PCA), they were strongly cultivar-dependent in apple and mainly season-dependent in pear (Supplementary Figure S1). In more detail, Fuji apples were characterized by higher soluble sugar levels than the other varieties and Granny Smith apples featured lower pH and sugar levels but greater hardness. Among the pear samples, fruits from the 2014 season were harder with lower soluble sugar levels (Decana del Comizio, Kaiser and Abate Fetel cultivars). Cultivar-specific clustering among the apple samples confirmed they were collected at a homogeneous ripening stage. In contrast, the clustering of pears mainly according to the growing season could be a typical feature of pears or may indicate a problem with the sampling method.

### 3.2. Targeted Exploration of the Apple and Pear Metabolome (NMR and HPLC-DAD)

**Figure 2:**
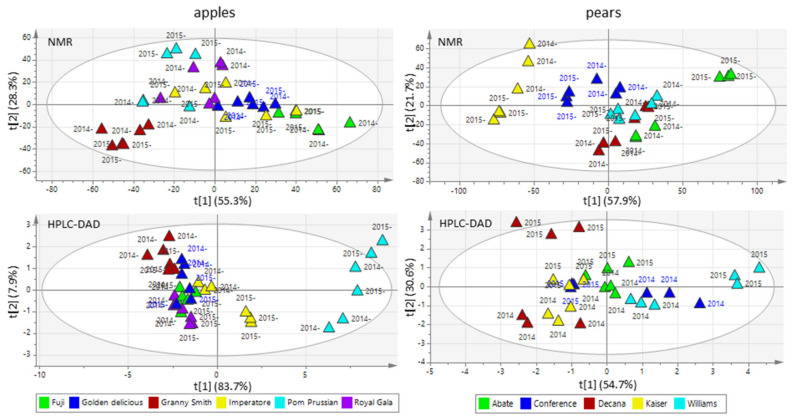
Score plots from the unsupervised PCA of apples (**left**) and pears (**right**) based on quantitative data from NMR (**top row**) and HPLC-DAD analysis (**bottom row**).

Targeted analysis of the six apple and five pear cultivars by NMR and HPLC-DAD allowed the quantification of all major primary and secondary metabolites (Supplementary Table S1). PCA (Figure 2) and O2PLS-DA (Supplementary Figure S2) based on these metabolic profiles showed that the apples and pears could be partially clustered by cultivar and growing season. O2PLS-DA loadings allowed to evaluate the importance of each metabolite on the observed clustering, as is shown in *pq(corr)1* vs. *pq(corr)2* loading plots (Supplementary Figure S2).

**Figure 3:**
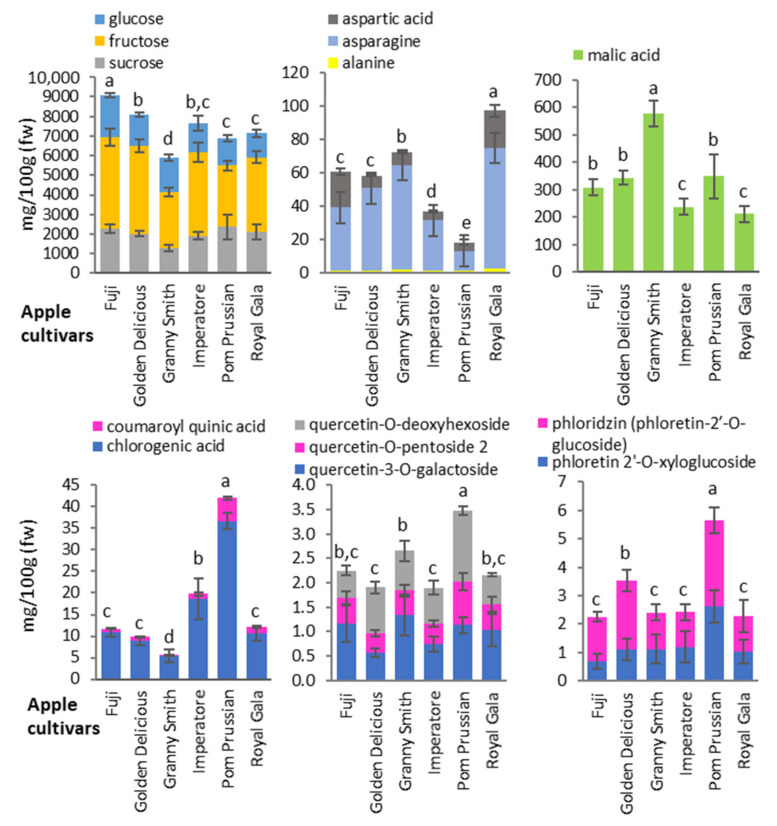
Quantities of the main primary and secondary metabolites in apple cultivars. Primary metabolites were quantified by NMR spectroscopy and secondary metabolites by HPLC-DAD. Data are expressed as mg/100 g fresh weight (fw) and are means of *n* = 3 samples representing two growing seasons ± standard deviations. Different letters on bars indicate that means referred to the sum of the molecules presented in each graph are significantly different among the apple cultivars according to post-hoc SNK-ANOVA (*p* < 0.05).

In detail, the Granny Smith apples were strongly characterized by higher malic acid and lower sugar levels, and weakly characterized by low levels of the main secondary metabolites. Fuji apples were characterized by high sugar levels (especially glucose and fructose) and low levels of secondary metabolites. Pom Prussian, an ancient cultivar local to northern Italy, was characterized by the highest level of secondary metabolites and low levels of amino acids. Royal Gala accumulated high levels of amino acids. Golden Delicious accumulated low levels of quercetin-3-O-galactoside and higher levels of phloridzin. Finally, Imperatore apples were characterized by a general low level of amino acids, malic acid, and flavonoids (Figure 3, Supplementary Table S1 and Supplementary Figure S2).

**Figure 4:**
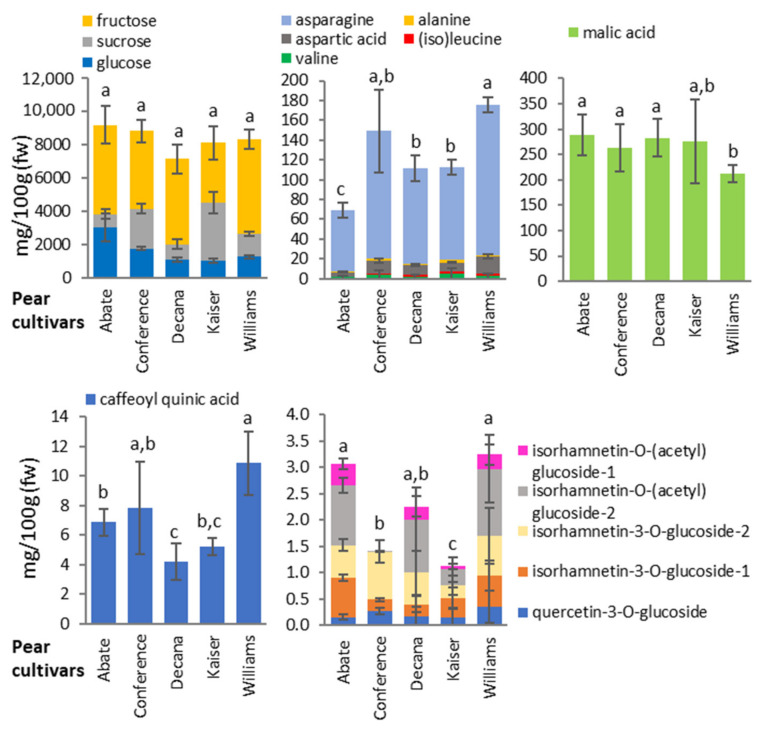
Quantities of the main primary and secondary metabolites in pear cultivars. Primary metabolites were quantified by NMR spectroscopy and secondary metabolites by HPLC-DAD. Data are expressed as mg/100 g of fresh weight (fw) and are means *n* = 3 samples representing two growing seasons ± standard deviations. Different letters on bars indicate that means referred to the sum of the molecules presented in each graph are significantly different among the pear cultivars according to post-hoc SNK-ANOVA (*p* < 0.05).

Among the pear cultivars, Abate Fetel was characterized by low levels of amino acids and high levels of glucose and flavonoids. Conference was characterized by low flavonoid levels and the absence of acetylated isorhamnetin, Decana del Comizio by low levels of sugar (especially sucrose) and caffeoyl quinic acid, Kaiser by abundant sucrose but low levels of flavonoids, and Williams by high levels of amino acids and secondary metabolites in general but low levels of malic acid. Interestingly, the sugar profile (mainly sucrose, fructose and glucose) was highly cultivar-dependent, with Abate Fetel and Decana del Comizio in particular containing low levels of sucrose (Figure 4, Supplementary Table S1 and Supplementary Figure S2).

The effect of the growing season on each cultivar was explored by PCA using the whole HPLC-DAD and NMR data matrix (Supplementary Figure S3). As for the fruit quality features (°Brix, pH, and hardness), the effect of growing season was more pronounced in pears, but a clear effect was also observed in most of the apple cultivars. In both species, the growing season effect was cultivar-specific.

### 3.3. Untargeted Metabolomic Analysis of Apple and Pear Cultivars (HPLC-ESI-MS)

HPLC-DAD only allows the detection of major secondary metabolites, so we also carried out untargeted HPLC-ESI-MS analysis. This revealed 392 and 385 features (*m*/*z* and retention time pairs) in apples and pears, respectively (Supplementary File S1), and allowed the putative annotation of 99 *m*/*z* features in apples and 63 in pears, including hydroxycinnamic and hydroxybenzoic acids, flavonoids, flavan-3-ols, procyanidins, chalcones, and their isotopes and adducts. HPLC-ESI-MS indicated the presence of many minor metabolites present at levels too low for detection by HPLC-DAD. We were also able to quantify the flavan-3-ols catechin and epicatechin and various procyanidins, with polymerization degrees of 2–6. These could not be identified and quantified by HPLC-DAD because the elution peaks overlapped with those of other metabolites.

**Figure 5:**
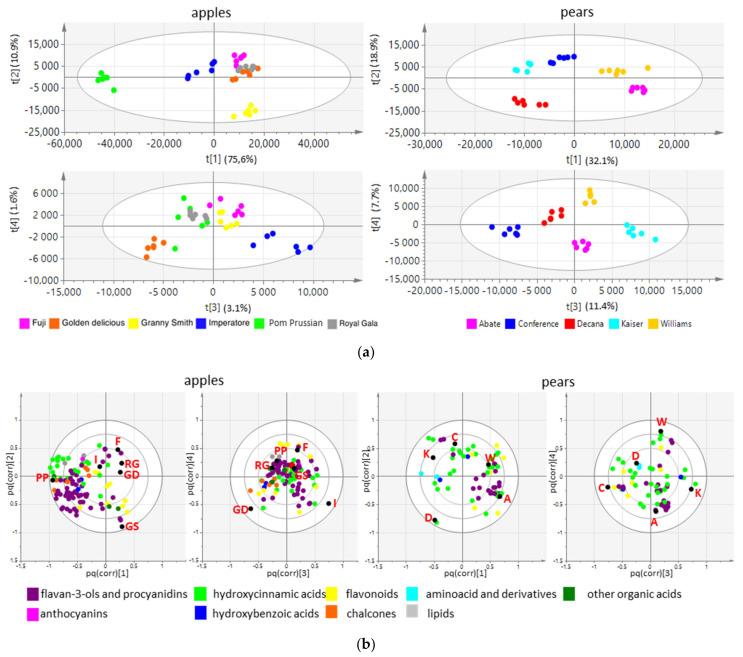
Supervised O2PLS-DA of apples and pears based on HPLC-ESI-MS data matrices. (**a**) Apple (**left**) and pear (**right**) score plots, with color dots representing different cultivars as defined in the key. (**b**) Apple (**left**) and pear (**right**) loading plots, with color dots representing different metabolite classes as defined in the key. Abbreviations for cultivars: Fuji (F), Golden Delicious (GD), Granny Smith (GS), Imperatore (I), Pom Prussian (PP), Royal Gala (RG); Abate (A), Conference(C), Decana del Comizio (D), Kaiser (K), and Williams (W).

The apple and pear data matrices (Supplementary File S1) were explored by O2PLS-DA (Figure 5). This not only confirmed the HPLC-DAD results, but also revealed the strong accumulation of flavan-3-ols and procyanidin in the Pom Prussian apple cultivar and (to a lesser extent) in the Abate Fetel pear.

### 3.4. Relationships between Metabolites and Antioxidant Activity

**Figure 6:**
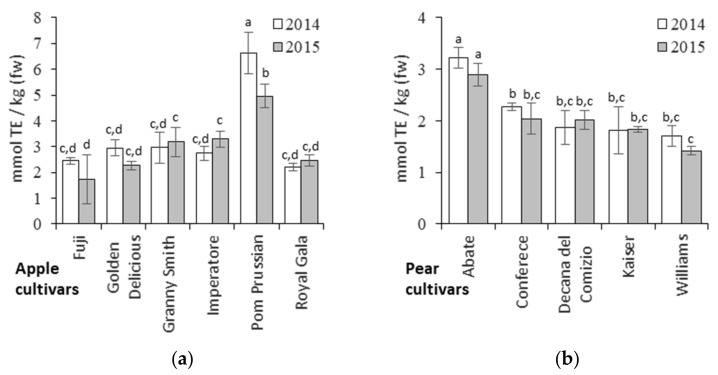
FRAP assay to measure the antioxidant activity of different apple (**a**) and pear (**b**) cultivars in different seasons. TE = Trolox equivalents. Data are means ± standard deviations of n = 3 samples. Different letters on bars indicate that means are significantly different according to post-hoc SNK-ANOVA (*p* < 0.05).

The antioxidant activity of the apples and pears was determined using a FRAP assay (Figure 6 and Supplementary File S1) revealing a strong cultivar-specific effect. Among the pears, the Abate Fetel cultivar showed the highest antioxidant activity, while Pom Prussian was the more active apple cultivar, far exceeding the activity of any other apple or pear cultivars.

**Figure 7:**
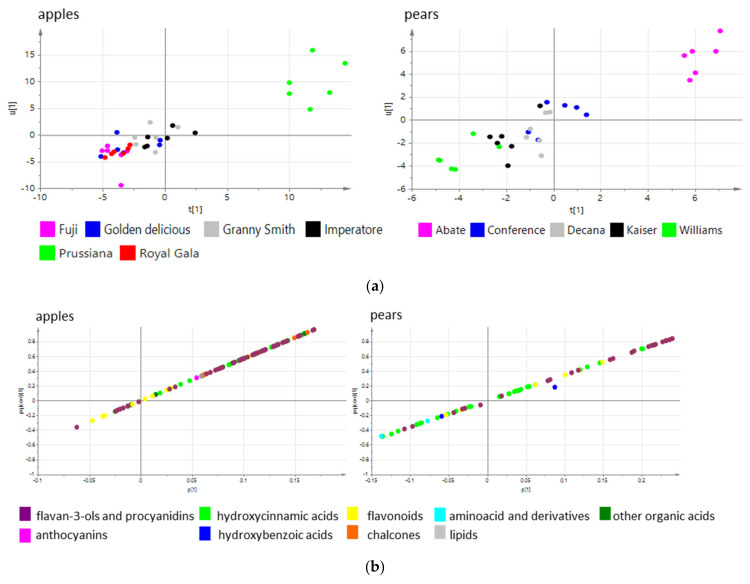
Supervised OPLS-DA of HPLC-ESI-MS data matrices for apple and pear cultivars to investigate the relationship between the antioxidant activity and the metabolomic profiles. (**a**) t1 versus u1 score plot; (**b**) p1 versus pq(corr)1 loading plot.

The relationship between antioxidant activity and the metabolomic profiles of the samples was investigated by OPLS-DA (Figure 7).

The purpose of OPLS-DA is to find joint variation between two groups of variables, the X variables (here the relative abundance of metabolites determined by HPLC-ESI-MS) and the Y variables (here the antioxidant activity determined in the FRAP assay), eliminating any variation in X that is orthogonal and thus not correlated to Y. The score plots (Figure 7a) separated the apple and pear cultivars by antioxidant activity, with Pom Prussian and Abate Fetel clearly distinguished from the other cultivars. The loading plots (Figure 7b) indicate the metabolites (X-variables) that co-vary with antioxidant activity, expressed as pq(corr) values. These quantify the correlation between p representing the X variables (metabolites) and q representing the Y variable (antioxidant activity). In apple, the metabolites that correlated with antioxidant activity were the chalcones and flavan-3ols/procyanidins. In pear, the metabolites that correlated with antioxidant activity were the flavan-3ols/procyanidins and some hydroxycinnamic acids. The pq(corr) values of the annotated metabolites are reported in Supplementary File S1. Among the flavan-3ols, catechin and epicatechin were abundant in the apple and pear cultivars, but epicatechin showed a stronger correlation with antioxidant activity.

## 4. Discussion

The metabolic composition and/or antioxidant activity of apples has been reported in many studies, reflecting the strong consumer interest in this fruit, but there are far fewer equivalent studies of pears. One challenge when comparing these studies is the differences in experimental planning and sampling methods. For example, quantities may be reported as a proportion of fresh weigh [18] or dried weight [19], samples may represent the whole fruit (peel and flesh or, more correctly, the cortex of the false fruit) [20] or separated peel and/or flesh [18,21], samples may consist of individual fruits [19] or pools [22], and in the latter case the sample may be a single pool [21] or more than one independent pool [23], and samples may cover one [24] or more growing seasons [25]. Agronomic traits such as fruit weight, acidity and the soluble solids content are reported in some studies but not others. Only rarely is a sampling plan specifically designed to represent a wide zone [22].

Given that apples and pears are generally consumed as fresh fruits with skins, we decided to report quantities as a proportion of fresh weight and to analyze a fruit portion that included both peel and flesh. The latter is important because the skin contributes significantly to the nutritional value of apples and pears in the diet. The experimental plan included many orchards representing a wide zone and we collected three independent large pools (50 fruits in each) with sampling across two growing seasons. We evaluated some agronomic traits to ensure a consistent ripening stage during collection. Furthermore, we complemented the targeted analysis of major metabolites with the untargeted analysis of a larger range of metabolites. Six apple cultivars and five pear cultivars were compared.

We found that the agronomic traits were cultivar-specific in apple but growing season-dependent in pear. This indicated that the ripening stage of the apple samples was comparable across the two growing seasons but this was not the case for the pear samples, possibly indicating seasonal effects but we cannot exclude the possibility of sampling errors relating to the stage of ripening.

Given the diverse experimental approaches used in earlier studies, the comparison of different reports is challenging. However, in the case of Golden Delicious apples (a widely grown cultivar that has been subject to detailed characterization), we compared the levels of major specialized metabolites and found that our study largely agreed with previous reports. For example, the levels of chlorogenic acid and phloretin xyloglucoside were consistent with those reported by Lee [20] and Vrhovsek [22], and the levels of coumaroyl quinic acid, phoridzin, quercetin-O-rhamnoside and quercetin-O-xylosides were similar to those reported by Vrhovsek [22]. However, quercetin-3-O-galactoside levels reported by both authors were approximately three times higher than the results we observed.

Fruits and vegetables provide a rich source of phytochemicals, many with potent antioxidant activity in vitro. For example, polyphenols such as chlorogenic acid, phloretin and isorhamnetin derivatives (abundant in apples and pears) feature aromatic rings decorated with hydroxyl groups that are good electron and hydrogen donors, allowing them to act as efficient scavengers of reactive oxygen species (ROS) [26,27,28]. The compounds with antioxidant activity are necessary to maintain redox homeostasis. Curiously, while redox homeostasis has been studied extensively in leaves, the fundamental knowledge of these processes in fruits, which accumulate large amounts of antioxidants, is still fragmentary, as recently reviewed by Decros et al. [6].

Diets rich in plant-derived polyphenols have been shown to reduce oxidative stress and inflammation, thus lowering the risk of non-communicable diseases based on a chronic inflammatory state (e.g., cancer, neurodegenerative diseases, cardiovascular diseases, obesity and diabetes) [29]. The health-promoting effects of such diets depend on the content, composition, and bioavailability of polyphenols [30] and other phytochemicals that may act synergistically, as shown for apple polyphenols and dietary fiber [31]. This is the basis of recommendations to include 5–7 daily servings of fruit and vegetables in the diet, ensuring an adequate intake of different classes of these beneficial compounds [32]. The inclusion of daily serving of Golden Delicious apples in older people lacking a Mediterranean-style diet is positively correlated with a reduced risk of colorectal cancer [33]. Similarly, an observational study in Australia reported the dose-dependent reduction of cancer risk in elderly women when apples were included in the diet, with the risk reduced by ~30% for diets supplemented with more than 100 g of apples per day [34].

The composition of fruits in terms of specialized metabolites depends on both genetic and environmental factors, although the former generally has a stronger effect. There are up to 3000 apple varieties and 2000 pear varieties worldwide, but only a few of these are cultivated commercially, restricting the choice for consumers [4,35]. The continual strong selection pressure imposed to breed elite cultivars with visually attractive and delicious fruits that withstand long storage periods [36,37] has reduced the content of bioactive compounds, including polyphenols [22,23,38,39]. In contrast, a large body of literature reports much higher level of these polyphenols in ancient and poorly exploited varieties (see for instance [5,40,41,42,43]). For example, the chlorogenic acid content of Golden Delicious, Granny Smith and Royal Gala apples was reported as ~9.5, ~3.2 and ~6.5 mg/100 g fw, respectively, and the same varieties accumulated ~4.5, ~1.6 and ~1.2 mg/100 g fw of phloridzin, and ~3.3, ~1.8 and ~3.2 mg/100 g fw of quercetin 3-O-galactoside [44]. Our results are in broad agreement (Supplementary Table S1). Another study reported the chlorogenic acid content of Royal Gala, Golden Delicious and Fuji apple skins as ~12, ~6 and ~16 mg/100 g fw, and the same varieties accumulated ~1.4, ~0.8 and ~1.62 mg/100 g fw of phloridzin [35]. Compared to these regular cultivars, the analysis of nine ancient apple varieties revealed that the chlorogenic acid content was three times higher (~28 mg/100 g fw) in variety Dal Doc, and that the phloridizin content ranged from twice as high (~3 mg/100 g fw in variety Limoncei) to 10-fold higher (~14 mg/100 g fw in variety Di Corone) [35]. In our study, we observed that fruits of the ancient variety Pom Prussian accumulate much higher levels of chlorogenic acid (~35 and ~38 mg/100 g fw in 2014 and 2015, respectively) and phloridzin (~3 mg/100 g fw) than the previously described traditional varieties. In fact, values of chlorogenic acid and phloridzin in Dal Doc, Limoncei and Di Corone varieties are referred to skin tissue content, whereas levels in Pom Prussian are referred to skin and pulp tissues. Considering that skin cells are noted to accumulate more secondary metabolites than pulp, this means that Pom Prussian can be an excellent healthy choice due to the high content of secondary metabolites. Additionally, the relative levels of procyanidins and flavan-3-ols were much higher in this cultivars compared with the others. Accordingly, this cultivar showed a much higher antioxidant activity in vitro, and the analysis of covariation of metabolite levels and antioxidant activity attributed this higher activity mainly to the chalcones and the procyanidin and flavan-3-ols. The superior nutritional profile of the ancient apple varieties, largely described in the scientific literature and confirmed in this report for a previously not investigated variety, Pom Prussian, depends mainly on two factors: first, the selection of the more largely consumed apple varieties simply did not take in account the nutritional profile, relying rather on appearance, post-harvest shelf-life and good taste; second, higher polyphenol level and, thus, better nutritional profile, were probably counter selected since they result in some characteristics, such as bitter and astringent taste and easy pulp browning, that may even be unwelcome [3,45,46,47].

We also found that Williams pears accumulate relatively low levels of chlorogenic acid (~9 mg/100 g fw) in both the peel and flesh tissues, similar to the levels reported in another recent investigation (~2 mg/100 g fw) [5]. However, traditional pear varieties accumulate higher levels of this metabolite, as reported in Sarikum pears (~89 and ~135 mg/100 g fw of chlorogenic acid in the flesh and peel, respectively) [5]. The consumption of apples and pears harvested from these underexploited varieties could therefore be used to improve the intake of dietary polyphenols.

In addition to the effect of genotype, the environment can influence the polyphenol content of fruits, albeit to a lesser extent. The polyphenol content in the skins of Granny Smith and Gold Rush apples was evaluated after harvesting fruits from the same plants located at the Agricultural Institute (Osijek, Croatia) in two growing seasons [25]. The results showed that procyanidin and dihydrochalcone levels remained stable regardless of the environmental conditions, whereas flavonols (quercetin derivatives) were more strongly affected [25]. Furthermore, Golden Delicious, Granny Smith and Starking fruits exposed to light and higher temperatures in the canopy, and harvested in two growing seasons, accumulated more phenolic compounds (measured using the Folin–Ciocalteu method) and showed greater antioxidant activity (determined using the ABTS assay) [48]. Moreover, the comparison of four apple cultivars grown in four geographically distinct locations in Chile revealed that apples from cooler areas and higher altitudes accumulated more polyphenols [49]. Similarly, four pear cultivars grown in two valleys in Pakistan accumulated different amounts of total phenolic compounds depending on the cultivation area [50]. Additionally, in our work fruits harvested in two different growing seasons showed slightly different metabolite profiles.

## 5. Conclusions

In conclusion, we have characterized the secondary metabolome of six apple and five pear cultivars, revealing that the local Pom Prussian apple and Abate Fetel pear have superior properties including high levels of antioxidant polyphenols and low levels of sucrose. Detailed analysis of the phenolic content of other apple and pear cultivars is necessary to identify which varieties accumulate the most polyphenols and could thus be recommended as constituents of a healthy diet. In this manner, consumers will be offered a greater choice and would be able to make informed decisions when selecting fruit with a more beneficial polyphenol profile. Ancient and underexploited varieties deserve greater attention given the extraordinary levels of polyphenols, as in the local apple variety Pom Prussian. We think that the growing attention to healthier foods should guide a new process of varietal development in apple and pear, starting from the vast heritage of the ancient varieties.

## Data Availability

Data is contained within the article or Supplementary Materials.

## Supplementary Materials

The following are available online at https://www.mdpi.com/article/10.3390/biology10050380/s1, Table S1: Quantities (mg/100 g fw) of the most abundant metabolites in apples and pears, Figure S1: Unsupervised PCA of agronomic data for apples and pears, Figure S2: Supervised O2PLS-DA of NMR and HPLC-DAD data matrices for apples and pears, Figure S3: PCA of NMR-HPLC/DAD combined data matrix, File S1: HPLC-ESI-MS data matrix (includes the peak area of each signal, the name of the annotated metabolites, FRAP antioxidant values, and the pq(corr) values of all the annotated metabolites based on OPLS-DA data).

## References

[1] USDA Fresh Apples, Grapes and Pear: World Markets and Trade. https://www.fas.usda.gov/data/fresh-apples-grapes-and-pears-world-markets-and-trade.

[2] Reiland H., Slavin J. (2015). Systematic review of pears and health. Nutr. Today.

[3] Persic M., Mikulic-Petkovsek M., Slatnar A., Veberic R. (2017). Chemical composition of apple fruit, juice and pomace and the correlation between phenolic content, enzymatic activity and browning. Lebensm. Wiss Technol..

[4] Brahem M., Renard C.M., Eder S., Loonis M., Ouni R., Mars M., Le Bourvellec C. (2017). Characterization and quantification of fruit phenolic compounds of European and Tunisian pear cultivars. Food Res. Int..

[5] Öztürk A., Demirsoy L., Demirsoy H., Asan A., Gül O. (2015). Phenolic compounds and chemical characteristics of pears (*Pyrus Communis* L.). Int. J. Food Prop..

[6] Decros G., Baldet P., Beauvoit B., Stevens R., Flandin A., Colombié S., Gibon Y., Pétriacq P. (2019). Get the balance right: ROS homeostasis and redox signalling in fruit. Front. Plant Sci..

[7] Kim E.S., Hong W.K. (2005). An apple a day ... does it really keep the doctor away? The current state of cancer chemoprevention. J. Natl. Cancer Inst..

[8] Lotito S.B., Frei B. (2004). Relevance of apple polyphenols as antioxidants in human plasma: Contrasting in vitro and in vivo effects. Free Radic. Biol. Med..

[9] Patocka J., Bhardwaj K., Klimova B., Nepovimova E., Wu Q., Landi M., Kuca K., Valis M., Wu W. (2020). Malus domestica: A Review on Nutritional Features, Chemical Composition, Traditional and Medicinal Value. Plants.

[10] Shinohara K., Ohashi Y., Kawasumi K., Terada A., Fujisawa T. (2010). Effect of apple intake on fecal microbiota and metabolites in humans. Anaerobe.

[11] Weichselbaum E., Wyness L., Stanner S. (2010). Apple polyphenols and cardiovascular disease—A review of the evidence. Nutr. Bull..

[12] Johnson S.A., Navaei N., Pourafshar S., Akhavan N.S., Elam M.L., Foley E., Clark E.A., Payton M.E., Arjmandi B.H. (2016). Fresh pear (*Pyrus communis*) consumption may improve blood pressure in middle-aged men and women with metabolic syndrome. FASEB J..

[13] Navaei N., Pourafshar S., Akhavan N.S., Foley E.M., Litwin N.S., George K.S., Hartley S.C., Elam M.L., Rao S., Arjmandi B.H. (2017). Effects of Fresh Pear Consumption on Biomarkers of Cardiometabolic Health in Middle-Aged and Older Adults with Metabolic Syndrome. FASEB J..

[14] Commisso M., Bianconi M., Di Carlo F., Poletti S., Bulgarini A., Munari F., Negri S., Stocchero M., Ceoldo S., Avesani L. (2017). Multi-approach metabolomics analysis and artificial simplified phytocomplexes reveal cultivar-dependent synergy between polyphenols and ascorbic acid in fruits of the sweet cherry (*Prunus avium* L.). PLoS ONE.

[15] Commisso M., Anesi A., Dal Santo S., Guzzo F. (2017). Performance comparison of electrospray ionization and atmospheric pressure chemical ionization in untargeted and targeted liquid chromatography/mass spectrometry based metabolomics analysis of grapeberry metabolites. Rapid Commun. Mass Spectrom..

[16] Commisso M., Negri S., Bianconi M., Gambini S., Avesani S., Ceoldo S., Avesani L., Guzzo F. (2019). Untargeted and targeted metabolomics and tryptophan decarboxylase in vivo characterization provide novel insight on the development of kiwifruits (*Actinidia deliciosa*). Int. J. Mol. Sci..

[17] Negri S., Lovato A., Boscaini F., Salvetti E., Torriani S., Commisso M., Danzi R., Ugliano M., Polverari A., Tornielli G.B. (2017). The induction of noble rot (*Botrytis cinerea*) infection during postharvest withering changes the metabolome of grapevine berries (*Vitis vinifera* L., cv. Garganega). Front. Plant Sci..

[18] Masi E., Taiti C., Vignolini P., Petrucci A.W., Giordani E., Heimler D., Romani A., Mancuso S. (2017). Polyphenols and aromatic volatile compounds in biodynamic and conventional ‘Golden Delicious’ apples (*Malus domestica* Bork.). Eur. Food Res. Technol..

[19] Łata B., Trampczynska A., Paczesna J. (2009). Cultivar variation in apple peel and whole fruit phenolic composition. Sci. Hortic..

[20] Lee K.W., Kim Y.J., Kim D.-O., Lee H.J., Lee C.Y. (2003). Major phenolics in apple and their contribution to the total antioxidant capacity. J. Agric. Food Chem..

[21] Chinnici F., Gaiani A., Natali N., Riponi C., Galassi S. (2004). Improved HPLC determination of phenolic compounds in cv. Golden Delicious apples using a monolithic column. J. Agric. Food Chem..

[22] Vrhovsek U., Rigo A., Tonon D., Mattivi F. (2004). Quantitation of polyphenols in different apple varieties. J. Agric. Food Chem..

[23] Kschonsek J., Wolfram T., Stöckl A., Böhm V. (2018). Polyphenolic compounds analysis of old and new apple cultivars and contribution of polyphenolic profile to the in vitro antioxidant capacity. Antioxidants.

[24] Xu Y., Fan M., Ran J., Zhang T., Sun H., Dong M., Zhang Z., Zheng H. (2016). Variation in phenolic compounds and antioxidant activity in apple seeds of seven cultivars. Saudi J. Biol. Sci..

[25] Lončarić A., Piližota V. (2014). Effect of variety, growing season and storage on polyphenol profile and antioxidant activity of apple peels. Food Health Dis..

[26] Li X., Chen B., Xie H., He Y., Zhong D., Chen D. (2018). Antioxidant structure–activity relationship analysis of five dihydrochalcones. Molecules.

[27] Xu J.-G., Hu Q.-P., Liu Y. (2012). Antioxidant and DNA-protective activities of chlorogenic acid isomers. J. Agric. Food Chem..

[28] Zuo A., Yanying Y., Li J., Binbin X., Xiongying Y., Yan Q., Shuwen C. (2011). Study on the relation of structure and antioxidant activity of isorhamnetin, quercetin, phloretin, silybin and phloretin isonicotinyl hydrazone. Free Radic. Antioxid..

[29] Koch W. (2019). Dietary polyphenols—important non-nutrients in the prevention of chronic noncommunicable diseases. A systematic review. Nutrients.

[30] Shahidi F., Ambigaipalan P. (2015). Phenolics and polyphenolics in foods, beverages and spices: Antioxidant activity and health effects—A review. J. Funct. Foods.

[31] Bondonno N.P., Bondonno C.P., Ward N.C., Hodgson J.M., Croft K.D. (2017). The cardiovascular health benefits of apples: Whole fruit vs. isolated compounds. Trends Food Sci. Technol..

[32] WHO Increasing Fruit and Vegetable Consumption to Reduce the Risk of Noncommunicable Diseases. https://www.who.int/elena/titles/fruit_vegetables_ncds/en/.

[33] Jedrychowski W., Maugeri U. (2009). An apple a day may hold colorectal cancer at bay: Recent evidence from a case-control study. Rev. Environ. Health.

[34] Hodgson J.M., Prince R.L., Woodman R.J., Bondonno C.P., Ivey K.L., Bondonno N., Rimm E.B., Ward N.C., Croft K.D., Lewis J.R. (2016). Apple intake is inversely associated with all-cause and disease-specific mortality in elderly women. Br. J. Nutr..

[35] Preti R., Tarola A.M. (2021). Study of polyphenols, antioxidant capacity and minerals for the valorisation of ancient apple cultivars from Northeast Italy. Eur. Food Res. Technol..

[36] Agnolet S., Ciesa F., Soini E., Cassar A., Matteazzi A., Guerra W., Robatscher P., Storti A., Baric S., Dalla Via J. (2017). Dietary elements and quality parameters of 34 old and eight commercial apple cultivars grown at the same site in South Tyrol, Italy. Erwerbs-Obstbau.

[37] Dalla Via J., Mantinger H. (2012). Agricultural research in the field of fruit growing in South Tyrol evolution and outlook. Erwerbsobstbau.

[38] Jakobek L., García-Villalba R., Tomás-Barberán F.A. (2013). Polyphenolic characterisation of old local apple varieties from Southeastern European region. J. Food Compost. Anal..

[39] Oszmiański J., Lachowicz S., Gamsjäger H. (2020). Phytochemical analysis by liquid chromatography of ten old apple varieties grown in Austria and their antioxidative activity. Eur. Food Res. Technol..

[40] Giomaro G., Karioti A., Bilia A.R., Bucchini A., Giamperi L., Ricci D., Fraternale D. (2014). Polyphenols profile and antioxidant activity of skin and pulp of a rare apple from Marche region (Italy). Chem. Cent. J..

[41] Panzella L., Petriccione M., Rega P., Scortichini M., Napolitano A. (2013). A reappraisal of traditional apple cultivars from Southern Italy as a rich source of phenols with superior antioxidant activity. Food Chem..

[42] Piccolo E.L., Landi M., Massai R., Remorini D., Conte G., Guidi L. (2019). Ancient apple cultivars from Garfagnana (Tuscany, Italy): A potential source for ‘nutrafruit’production. Food Chem..

[43] Sut S., Zengin G., Maggi F., Malagoli M., Dall’Acqua S. (2019). Triterpene acid and phenolics from ancient apples of Friuli Venezia Giulia as nutraceutical ingredients: LC-MS study and in vitro activities. Molecules.

[44] Valavanidis A., Vlachogianni T., Psomas A., Zovoili A., Siatis V. (2009). Polyphenolic profile and antioxidant activity of five apple cultivars grown under organic and conventional agricultural practices. Int. J. Food Sci. Technol..

[45] Holderbaum D.F., Kon T., Kudo T., Guerra M.P. (2010). Enzymatic browning, polyphenol oxidase activity, and polyphenols in four apple cultivars: Dynamics during fruit development. HortScience.

[46] Lea A.G., Arnold G.M. (1978). The phenolics of ciders: Bitterness and astringency. J. Sci. Food Agric..

[47] Vidal S., Francis L., Guyot S., Marnet N., Kwiatkowski M., Gawel R., Cheynier V., Waters E.J. (2003). The mouth-feel properties of grape and apple proanthocyanidins in a wine-like medium. J. Sci. Food Agric..

[48] Hamadziripi E.T., Theron K.I., Muller M., Steyn W.J. (2014). Apple compositional and peel color differences resulting from canopy microclimate affect consumer preference for eating quality and appearance. HortScience.

[49] Yuri J.A., Neira A., Quilodran A., Motomura Y., Palomo I. (2009). Antioxidant activity and total phenolics concentration in apple peel and flesh is determined by cultivar and agroclimatic growing regions in Chile. J. Food Agric. Environ..

[50] Hussain S., Masud T., Ali S., Bano R., Ali A. (2013). Some physico-chemical attributes of pear (*Pyrus communis* L.) cultivars grown in Pakistan. Int. J. Biosci..

