# Supplementary material for: Metabolomic Profiling and Antioxidant Activity of Fruits Representing Diverse Apple and Pear Cultivars
Source: Biology (Basel). 2021 Apr 28;10(5):380. doi: 10.3390/biology10050380 (PMC8145694; doi:10.3390/biology10050380)

## Figure S1 - Unsupervised PCA of agronomic data for apples and pears

*a) Score plots showing cultivars (top row) and growing seasons (bottom row)*

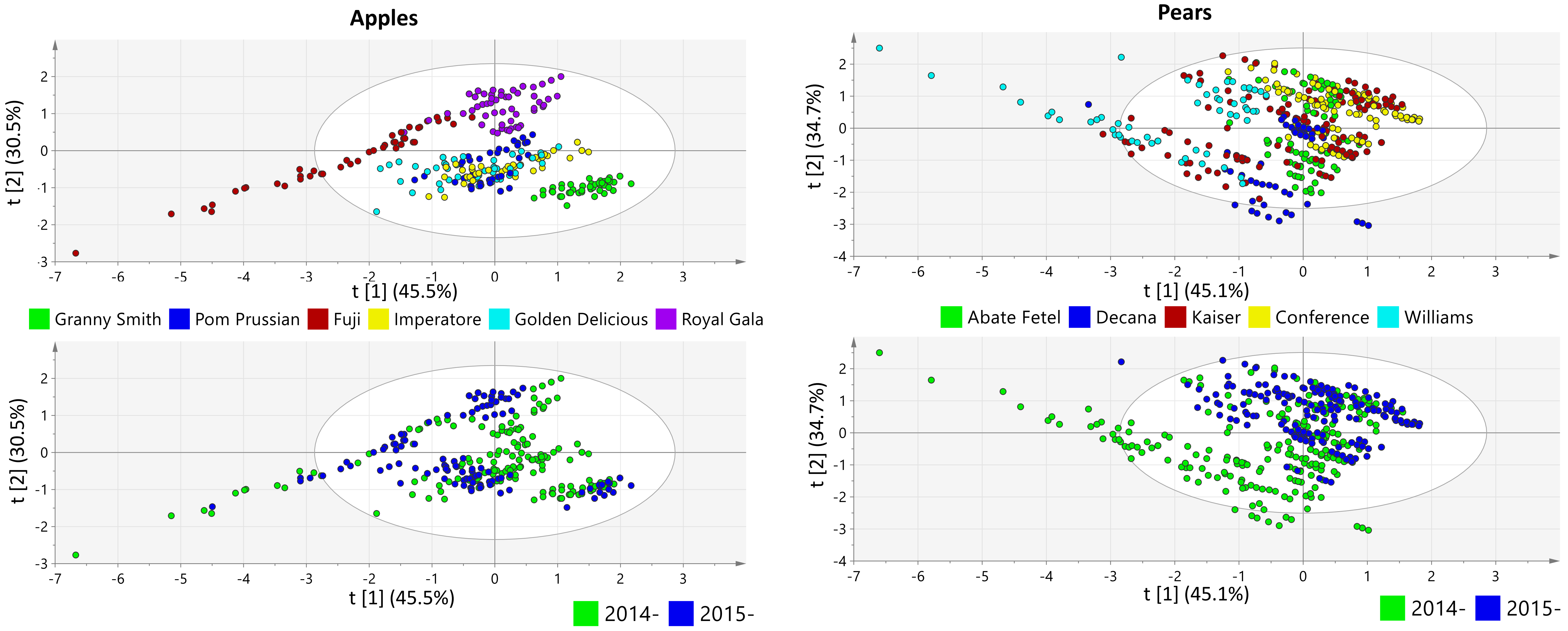


*b) Loading plots leading sample separation*

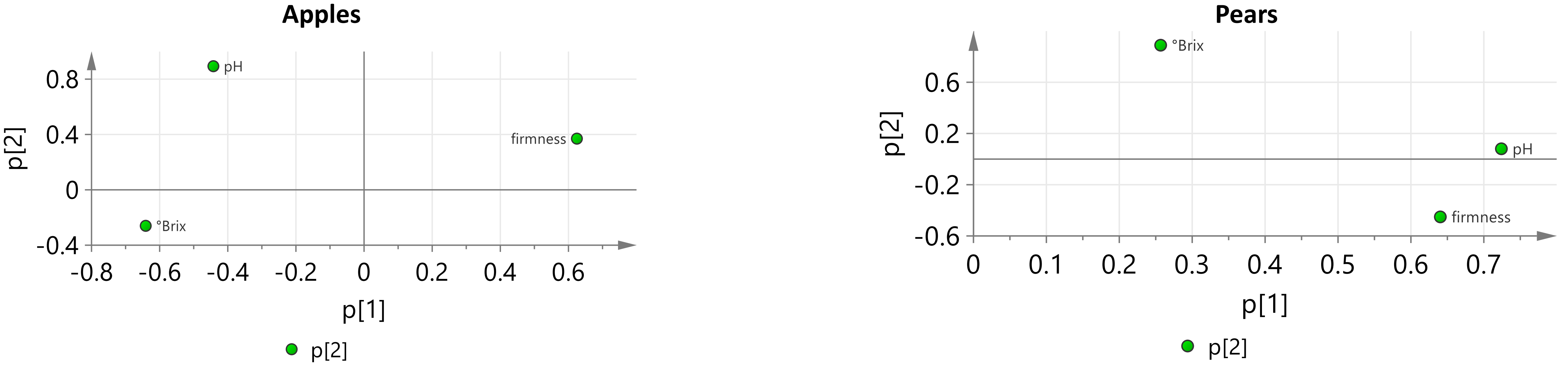

Supplement: Supplementary file 1 [file biology-10-00380-s001.zip › Figure S1.pdf]
