# Supplementary material for: Metabolomic Profiling and Antioxidant Activity of Fruits Representing Diverse Apple and Pear Cultivars
Source: Biology (Basel). 2021 Apr 28;10(5):380. doi: 10.3390/biology10050380 (PMC8145694; doi:10.3390/biology10050380)
Supplement: Supplementary file 1 [file biology-10-00380-s001.zip › Figure S2.pdf]

Figure S2. Supervised O2PLS-DA of NMR and HPLC-DAD data matrices for apples and pears

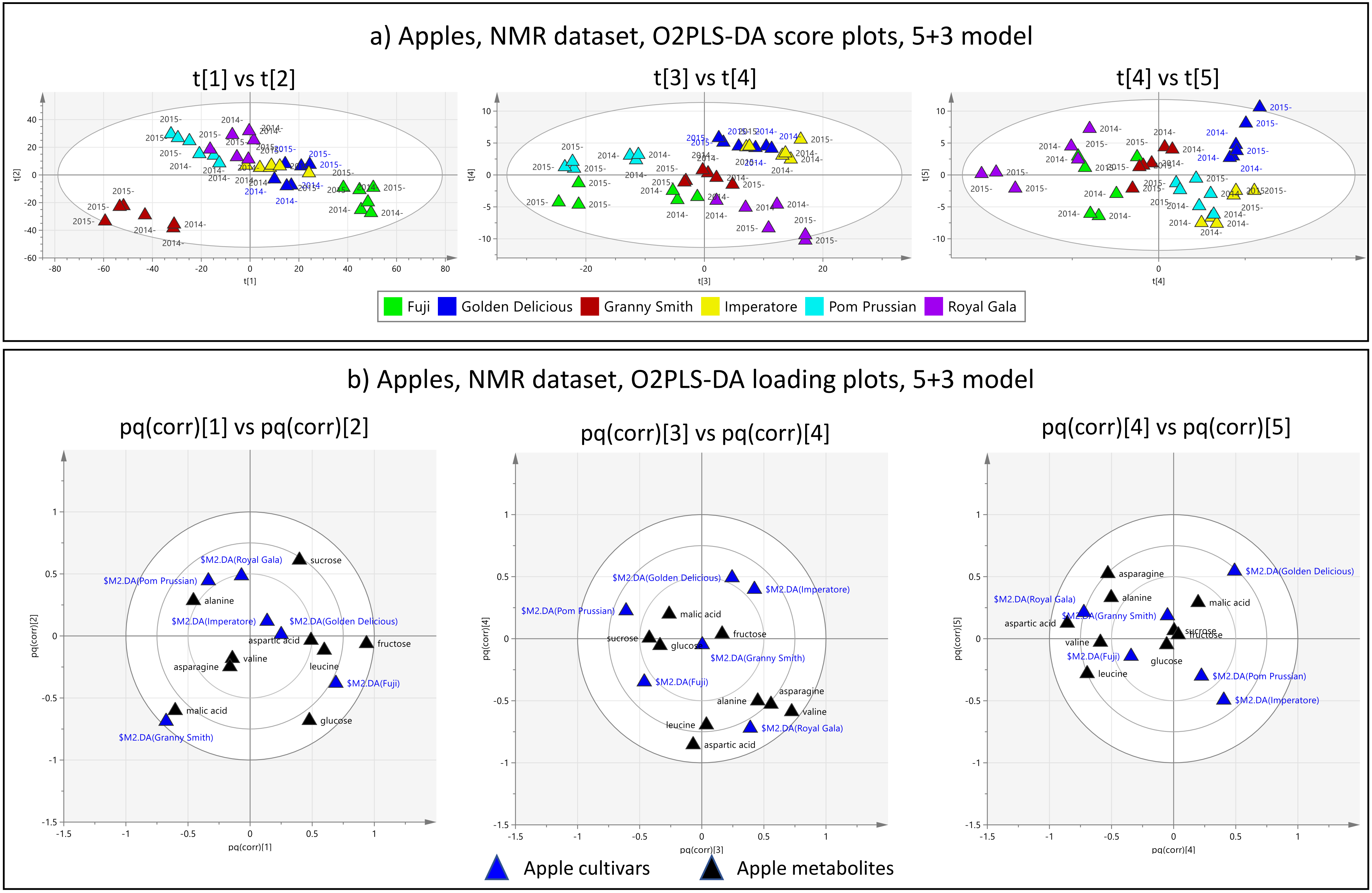

c) Apples, HPLC-DAD dataset, O2PLS-DA score plots, 5+2 model

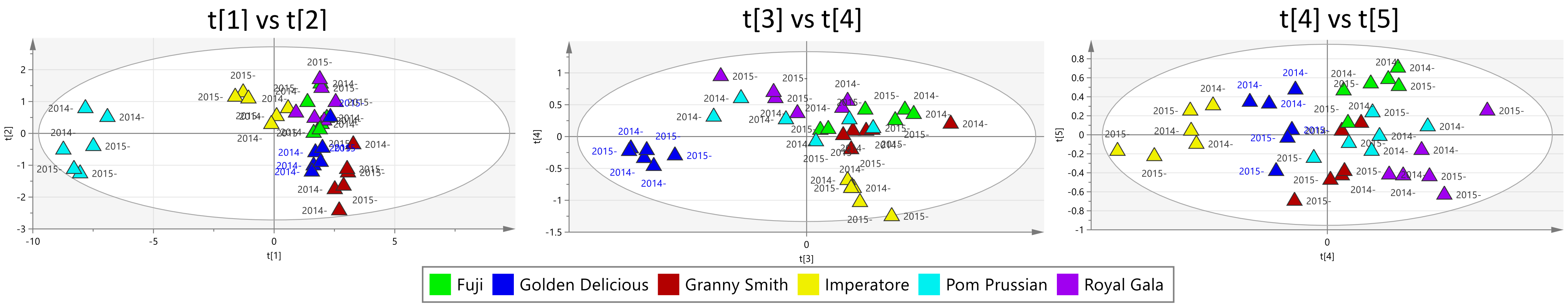

d) Apples, HPLC-DAD dataset, O2PLS-DA loading plots, 5+2 model

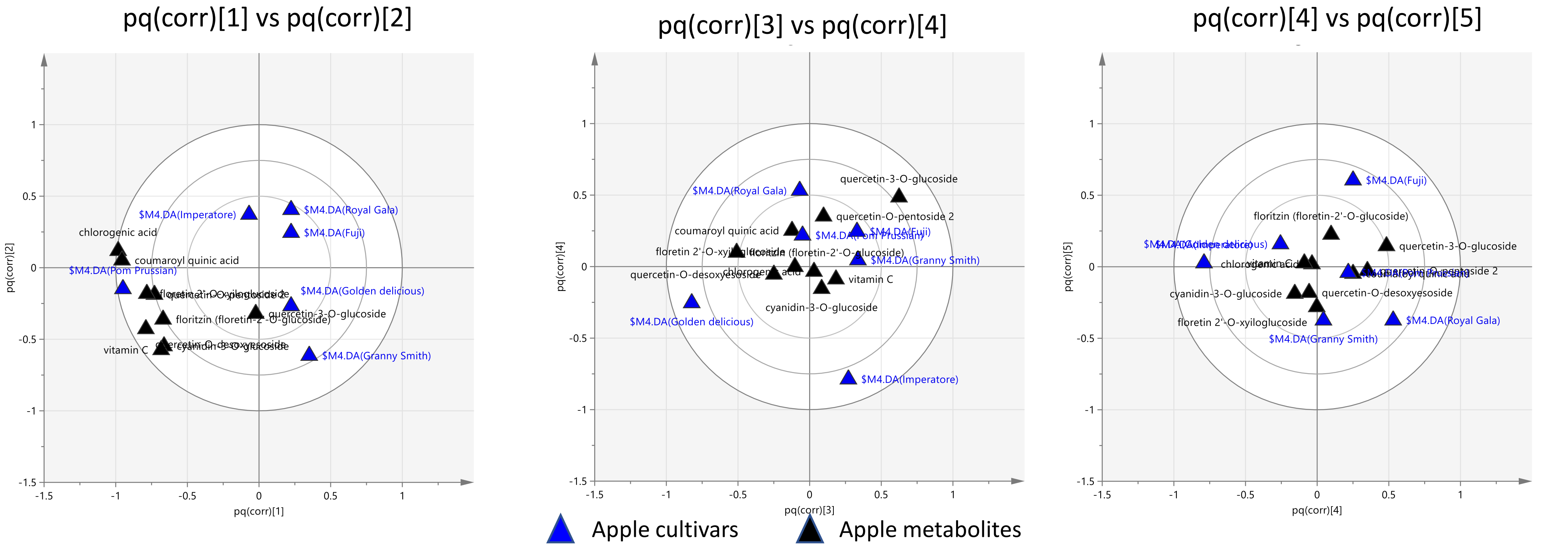

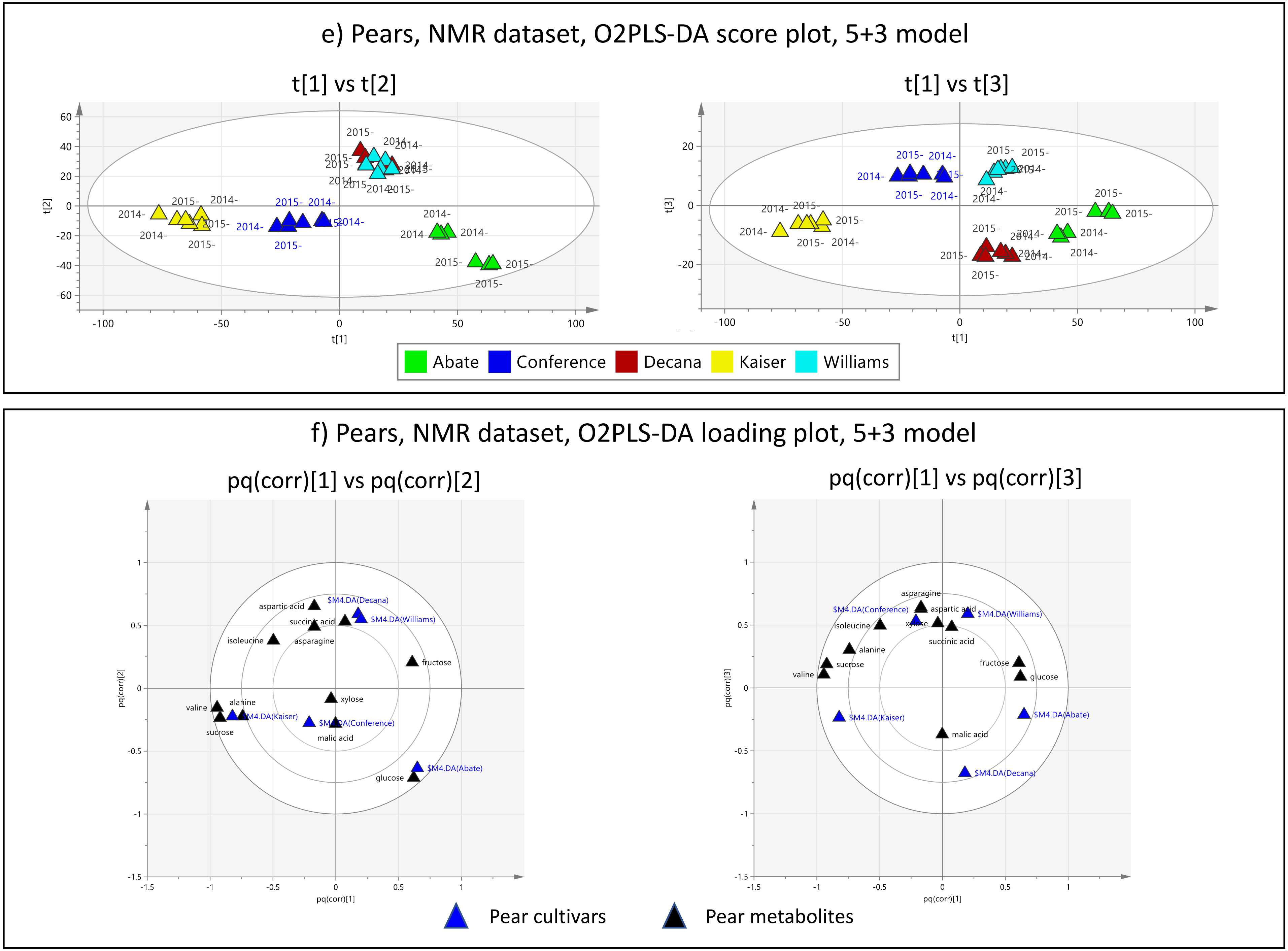
e) Pears, NMR dataset, O2PLS-DA score plot, 5+3 model
t[1] vs t[2]
t[1] vs t[3]
t[1]
t[2]
t[3]
Abate
Conference
Decana
Kaiser
Williams
f) Pears, NMR dataset, O2PLS-DA loading plot, 5+3 model
pq(corr)[1] vs pq(corr)[2]
pq(corr)[1] vs pq(corr)[3]
pq(corr)[1]
pq(corr)[2]
pq(corr)[3]
aspartic acid
succinic acid
asparagine
isoleucine
fructose
xylose
alanine
valine
sucrose
malic acid
glucose
$M4.DA(Decana)
$M4.DA(Williams)
$M4.DA(Kaiser)
$M4.DA(Conference)
$M4.DA(Abate)
Pear cultivars
Pear metabolites

g) Pears, HPLC-DAD dataset, O2PLS-DA score plot, 5+2 model

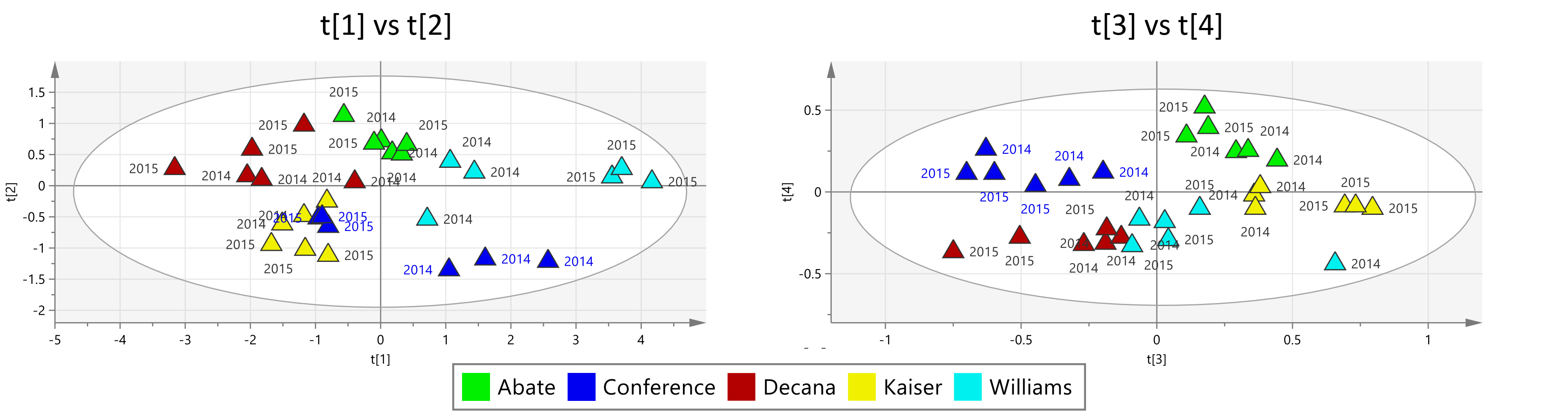

h) Pears, HPLC-DAD dataset, O2PLS-DA loading plot, 5+2 model

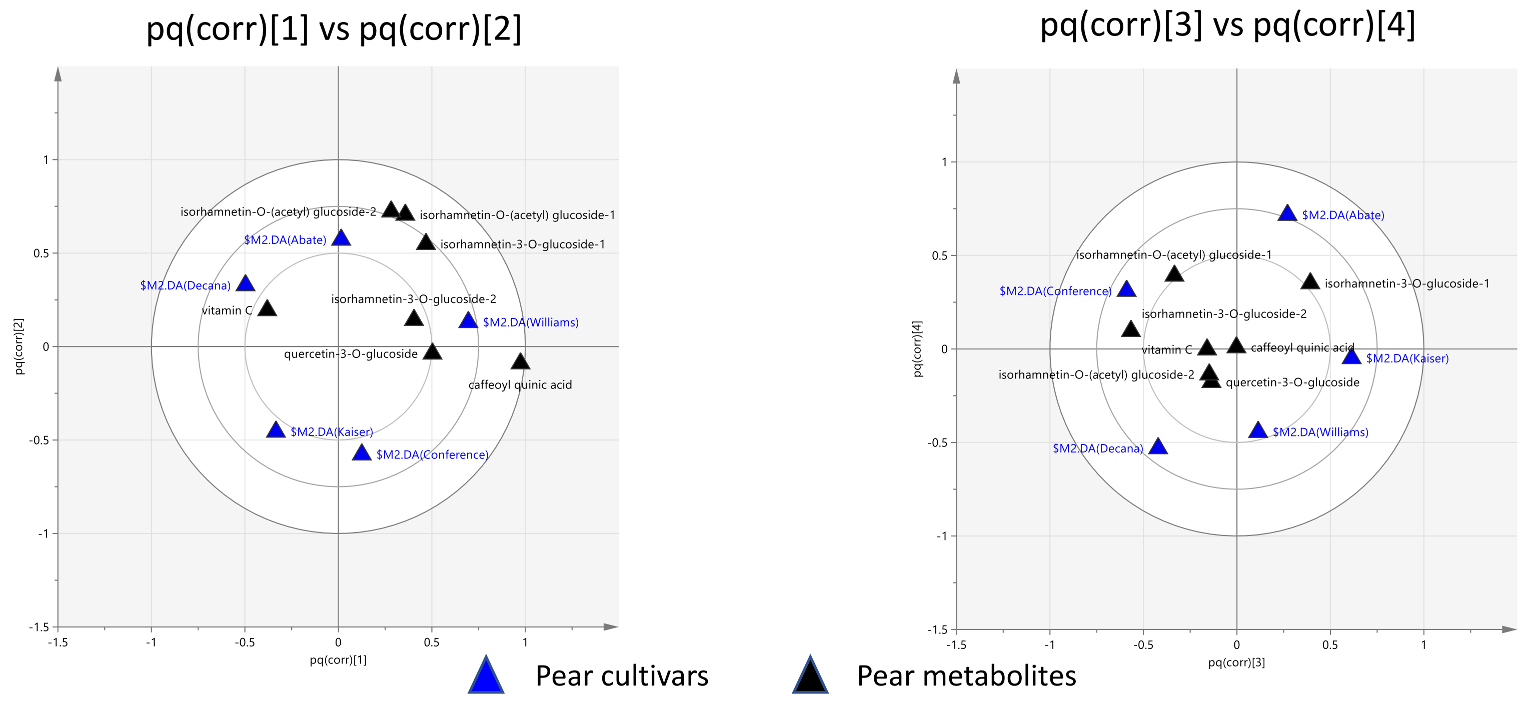

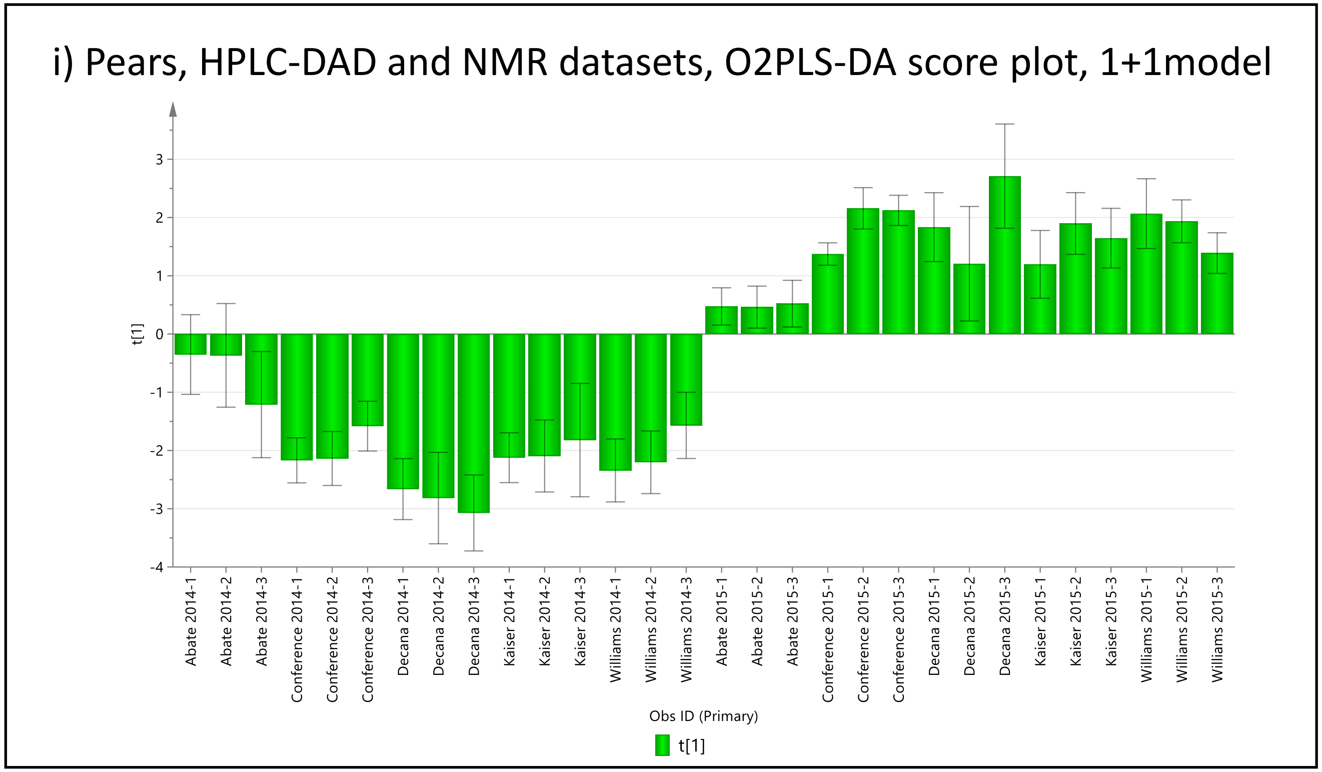
i) Pears, HPLC-DAD and NMR datasets, O2PLS-DA score plot, 1+1model
t[1]
Obs ID (Primary)
Abate 2014-1
Abate 2014-2
Abate 2014-3
Conference 2014-1
Conference 2014-2
Conference 2014-3
Decana 2014-1
Decana 2014-2
Decana 2014-3
Kaiser 2014-1
Kaiser 2014-2
Kaiser 2014-3
Williams 2014-1
Williams 2014-2
Williams 2014-3
Abate 2015-1
Abate 2015-2
Abate 2015-3
Conference 2015-1
Conference 2015-2
Conference 2015-3
Decana 2015-1
Decana 2015-2
Decana 2015-3
Kaiser 2015-1
Kaiser 2015-2
Kaiser 2015-3
Williams 2015-1
Williams 2015-2
Williams 2015-3
t[1]

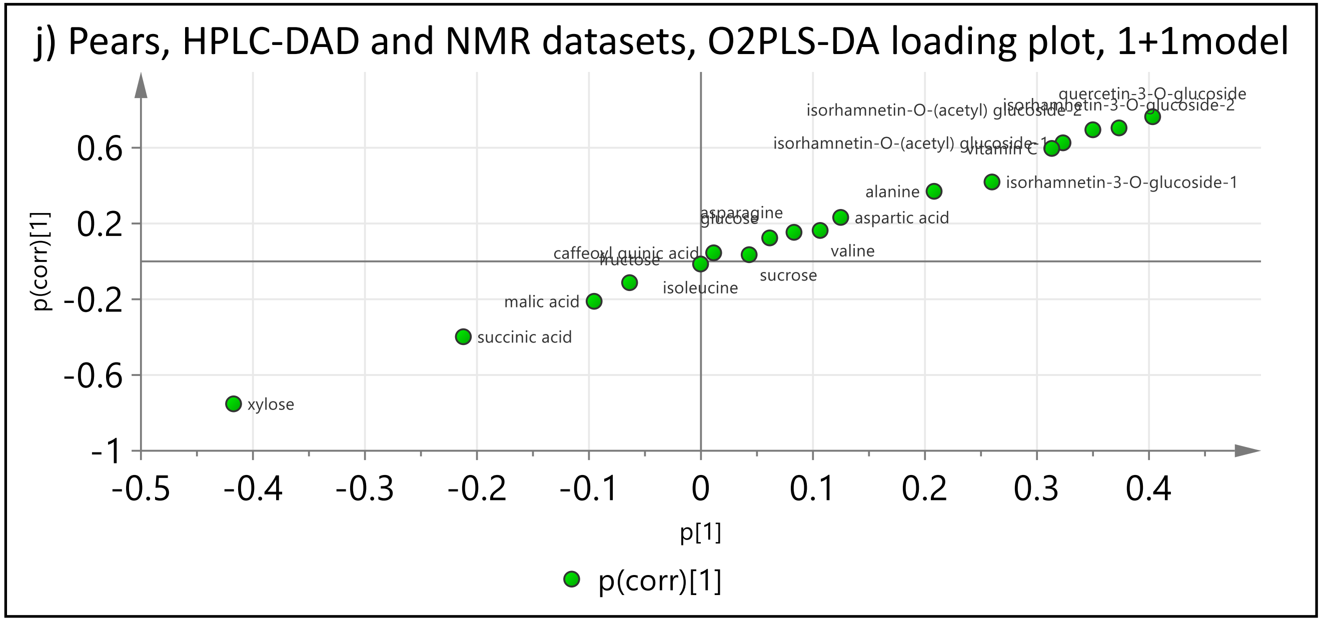
j) Pears, HPLC-DAD and NMR datasets, O2PLS-DA loading plot, 1+1model
p(corr)[1]
p[1]
quercetin-3-O-glucoside
isorhamnetin-3-O-glucoside-2
isorhamnetin-O-(acetyl) glucoside-2
isorhamnetin-O-(acetyl) glucoside-1
vitamin C
isorhamnetin-3-O-glucoside-1
alanine
aspartic acid
asparagine
glucose
valine
caffeoyl quinic acid
fructose
sucrose
isoleucine
malic acid
succinic acid
xylose
p(corr)[1]
