# Supplementary material for: Metabolomic Profiling and Antioxidant Activity of Fruits Representing Diverse Apple and Pear Cultivars
Source: Biology (Basel). 2021 Apr 28;10(5):380. doi: 10.3390/biology10050380 (PMC8145694; doi:10.3390/biology10050380)
Supplement: Supplementary file 1 [file biology-10-00380-s001.zip › Figure S3.pdf]

Figure S3. PCA of NMR-HPLC/DAD combined datamatrix

a) Apples

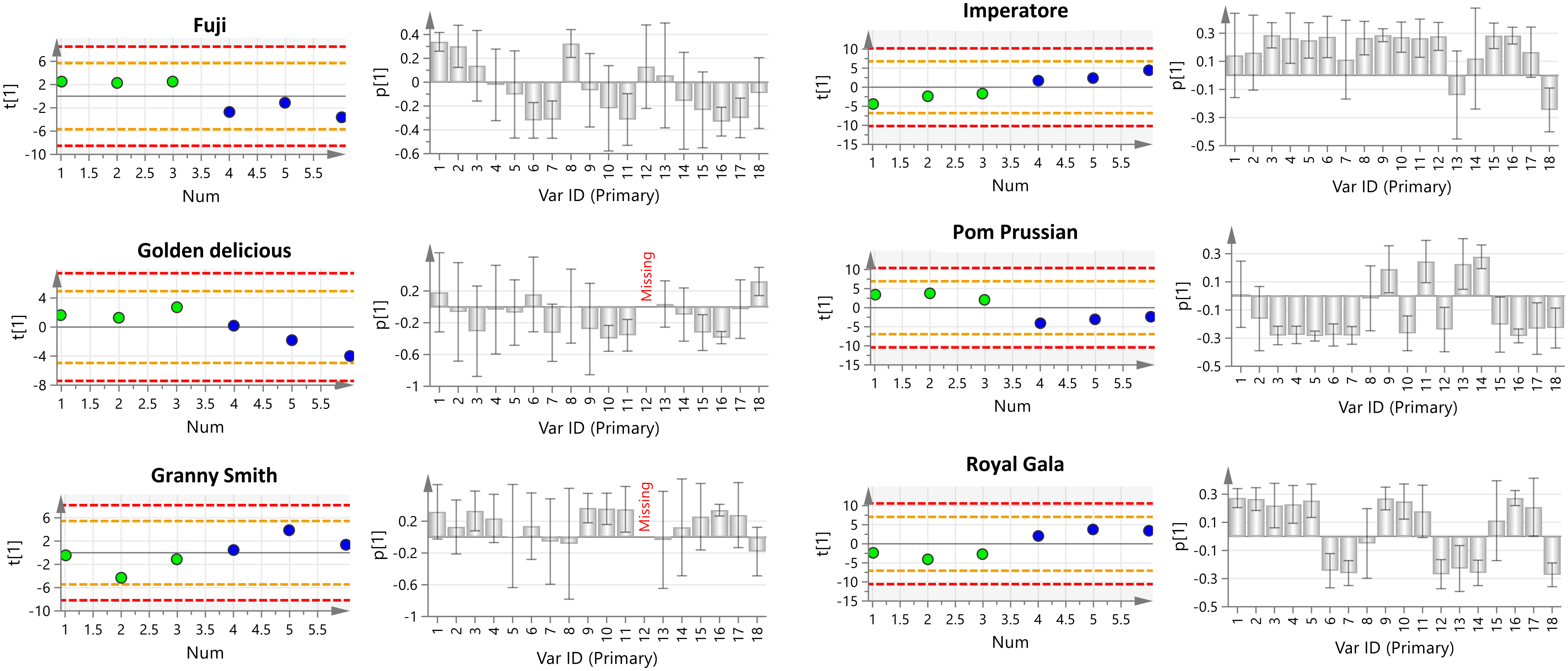

Var ID legend

1	valine	6	malic acid	11	coumaroyl quinic acid	16	phloretin 2'-O-xyloglucoside
2	leucine	7	sucrose	12	cyanidin-3-O-glucoside	17	phloridzin (phloretin-2'-O-glucoside)
3	alanine	8	fructose	13	quercetin-3-O-glucoside		
4	asparagine	9	glucose	14	quercetin-O-pentoside 2		
5	aspartic acid	10	chlorogenic acid	15	quercetin-O-pentoside 2		

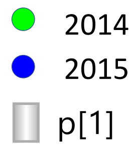

b) Pears

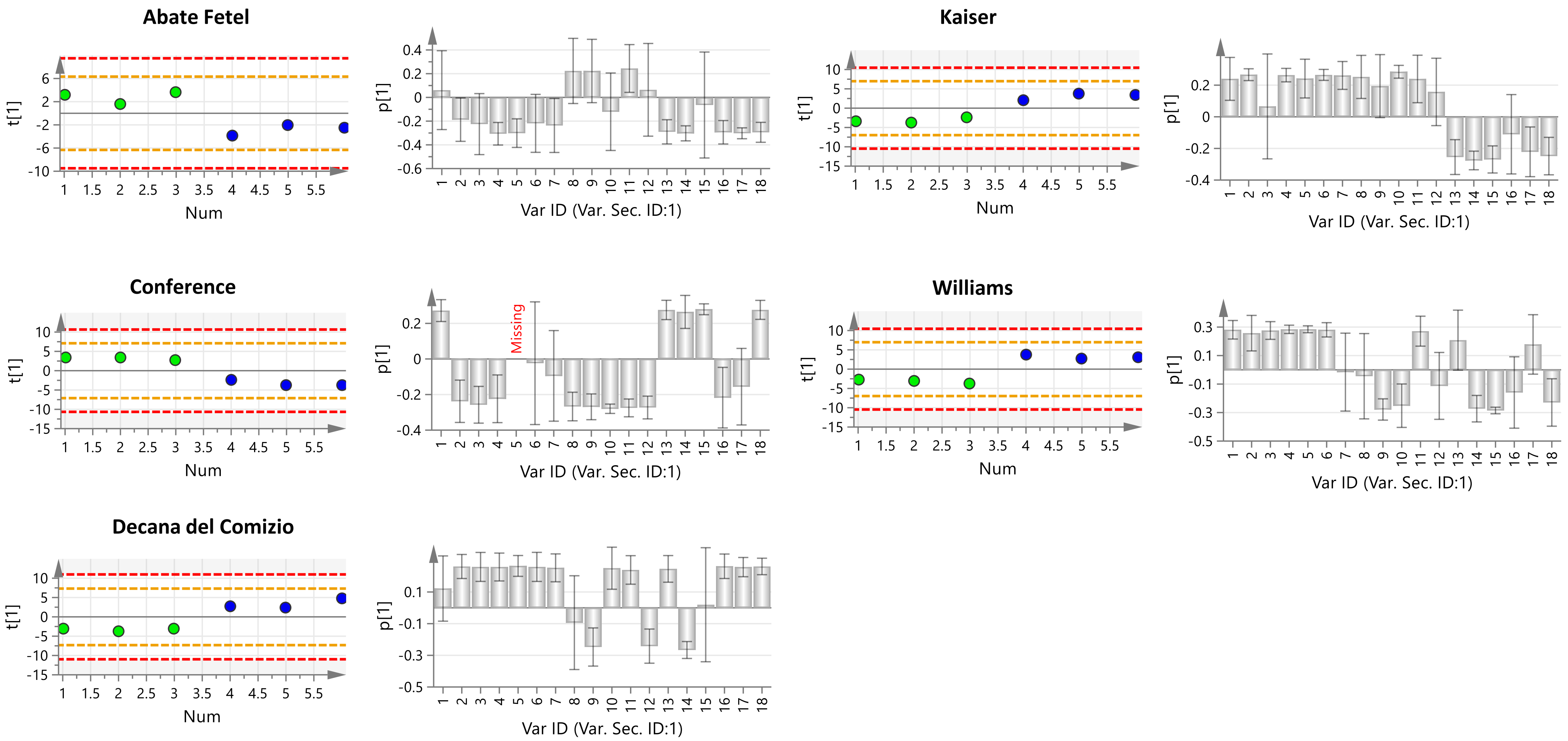

Var ID legend

1	caffeoyl quinic acid	6	isorhamnetin-O-(acetyl) glucoside-2	11	aspartic acid	16	glucose
2	quercetin-3-O-glucoside	7	unidentified	12	asparagine	17	sucrose
3	isorhamnetin-3-O-glucoside-1	8	valine	13	malic acid	18	fructose
4	isorhamnetin-3-O-glucoside-2	9	isoleucine	14	succinic acid		
5	isorhamnetin-O-(acetyl) glucoside-1	10	alanine	15	xylose		

2014
2015
p[1]
